# A Finite/Spectral Element Hybrid Method for Modeling and Band-Gap Characterization of Metamaterial Sandwich Plates

**DOI:** 10.3390/ma16031098

**Published:** 2023-01-27

**Authors:** Linzhongyang E, Zhijing Wu, Fengming Li, Guangping Zou

**Affiliations:** 1 College of Aerospace and Civil Engineering, Harbin Engineering University, Harbin 150001, China; 2 Institute of Systems Engineering, China Academy of Engineering Physics, Mianyang 621900, China

**Keywords:** elastic metamaterial sandwich plate, local resonance, band-gaps, spectral element method, finite element method, frequency response function

## Abstract

In this study, elastic metamaterial sandwich plates with axially deformed Timoshenko beam cores, considering both the out-of-plane and in-plane deformations of the face plates, are designed and the vibration band-gap properties are explored. The beam cores act as local resonators that can bear axial force, bending moment and shearing force. The finite element method (FEM) and the spectral element method (SEM) are combined to create the finite/spectral element hybrid method (FE-SEHM) for establishing the dynamic model and calculating the frequency response functions (FRFs) of the elastic metamaterial sandwich plate with axially deformed beam cores. It is observed that the metamaterial sandwich plate possesses both the axial and transverse vibration band-gaps of the beams, and the two kinds of band-gaps are independent. Compared with the metamaterial sandwich plates with rod cores, those with axially deformed beam cores have more extensive application ranges for vibration reduction.

## 1. Introduction

Elastic/mechanical/acoustic metamaterial structures [1,2,3], generally composed of periodically arranged substructures in the form of sandwich structures [4,5,6], have special mechanical properties. It is a research hotspot in the fields of mechanics, materials, physics and engineering in recent years [7,8,9,10]. Due to the characteristics of sub-wavelength sizes and dynamic equivalent parameters being different from those of traditional material structures, the metamaterial structures have a very broad application prospect in the fields of vibration reduction, sound absorption and wave direction control [11,12,13]. Researchers have designed many kinds of metamaterial structures such as rods [14,15], beams [16,17,18] and plates [19,20,21] which are composed of periodically distributed local resonators. A metamaterial plate is a two-dimensional periodic structure, which is mostly composed of continuous plates and periodic components such as holes and cylinders. Mass-spring systems, beams, four-bar linkages, and so on, are always used as the local resonators of metamaterial plates which possess the capacity for bearing loads and reducing vibrations.

The band-gap is the inherent property of elastic metamaterial structures [22,23,24], which can be used for passive control of structural vibration with the advantages of no external energy input, simple structure, strong reliability, good effect [25,26,27] and crack arrest and fracture resistance in the frequency region [28]. The frequency response functions (FRFs) are usually used to analyze the band-gap characteristics, and it is easy to directly judge whether the vibration can be effectively reduced within a certain frequency range by using the FRFs [29,30] or not. By designing the material and structural parameters, the positions and widths of band-gaps could be tuned to improve the vibration suppression ability of the metamaterial structures.

The band-gap analysis methods of metamaterial structures mainly include the plane wave expansion method [31,32], the transfer matrix method [33,34], the spectral element method (SEM) [35,36,37] and the finite element method (FEM) [38,39,40]. The plane wave expansion method is not suitable for processing three-dimensional (3D) structures and the calculation results are not easy to converge. It is difficult to deal with two-dimensional (2D) and 3D problems using the transfer matrix method. The SEM is not fit for solving complex structures. Additionally, it is time-consuming in the modeling process for the FEM and it is less accurate in dealing with high frequency problems. Each one of the FEM and SEM has its own limitation, but when they are combined to create the finite/spectral element hybrid method (FE-SEHM), they can make full use of their advantages. As the one-dimensional rods act as the local resonators and the irregular or complex matrices are analyzed by the SEM and FEM, respectively, the FE-SEHM can achieve unrestricted structural dimensions, convenient modeling, few element numbers and improved calculation accuracy and efficiency [41,42].

In this paper, on the basis of [41], wherein the core rods are assumed to have only axial deformations and only the transverse deformations of the cover plates are considered for the elastic metamaterial sandwich plates with rod cores, the FE-SEHM is further extended to investigate the band-gap characteristics of locally resonant elastic metamaterial sandwich plates with axially loaded Timoshenko beam cores considering the out-of-plane and in-plane vibrations of the thin face plates. The dynamic stiffness matrices of the beam elements and the plate elements are derived by the SEM and the FEM, respectively, and according to the nodal coordinate relationships, they are assembled to the whole dynamic stiffness matrix of the metamaterial sandwich plate. The FRFs of the elastic metamaterial sandwich plate are calculated. The axial vibration band-gaps of the beams are calculated and compared with those of the metamaterial plates with rod cores [41], and the transverse vibration band-gaps of the beams are also obtained and analyzed.

There are mainly the following three highlights and novelties for this study:

(1) The first one is the innovation of the research method, namely the FE-SEHM, developed for the structural modeling and band-gap characteristics analysis of metamaterial plates.
(2) The second one is that an elastic metamaterial sandwich plate with the axially deformed Timoshenko beam cores, considering both the out-of-plane and in-plane deformations of the face plates, is designed and investigated.
(3) The third one is that the metamaterial plate has flexural vibration band-gaps appearing near the flexural vibration natural frequencies of the beams, which uncouple with the axial vibration band-gaps.

## 2. Derivation of the Dynamic Stiffness Matrix

**Figure 1:**
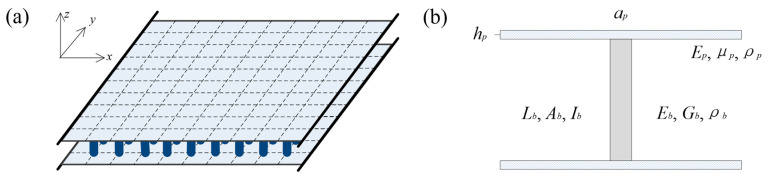
The metamaterial sandwich plate with axially deformed beam cores. (**a**) The 3D view, and (**b**) the unit-cell.

The metamaterial sandwich plate with axially deformed beam cores, as shown in Figure 1, is considered. The structure has three translational and two rotational degrees of freedom. The Cartesian coordinate system is displayed in Figure 1a in which the *x*, *y* and *z* axes denote the length, width and thickness directions of the sandwich plate. The beams acting as local resonators are periodically distributed between the two thin plates. The unit-cell consists one beam element and eight plate elements. As for the beams, *E_b_*, *G_b_* and *ρ_b_* represent the elastic modulus, the shear modulus and the mass density, and *L_b_*, *A_b_* and *I_b_* denote the length, the cross-sectional area and the moment of inertia. As for the plates, the elastic modulus, the Poisson’s ratio and the mass density are *E_p_*, *μ_p_* and *ρ_p_*, and the length, the width and thickness are *a_p_*, *b_p_* and *h_p_*.

### 2.1. Spectral Element Formulation of the Beam with Axial Deformation

For the free flexural vibration, the equations of motion of a Timoshenko beam element with uniform cross-section are written as [43]

(1) $$\kappa G_{b}\left(\frac{\partial^{2}v_{b}}{\partial z^{2}}-\frac{\partial \theta_{b}}{\partial z}\right)-\rho_{b}\frac{\partial^{2}v_{b}}{\partial t^{2}}=0,$$

(2) $$E_{b}I_{b}\frac{\partial^{2}\theta_{b}}{\partial z^{2}}+\kappa G_{b}A_{b}\left(\frac{\partial v_{b}}{\partial z}-\theta_{b}\right)-\rho_{b}I_{b}\frac{\partial^{2}\theta_{b}}{\partial t^{2}}=0,$$

where *κ* is the shear correction factor depending on the shape of the cross-section, *v_b_*(*z*, *t*) is the transverse displacement, *θ_b_*(*z*, *t*) is the rotation angle of the beam element and *t* is the time.

The equations of motion in the frequency domain can be obtained using the Fourier transformation, and they have the following formulae:

(3) $$\kappa G_{b}\left(\frac{\partial^{2}V_{b}}{\partial z^{2}}-\frac{\partial \Theta_{b}}{\partial z}\right)+\rho_{b}\omega^{2}V_{b}=0,$$

(4) $$E_{b}I_{b}\frac{\partial^{2}\Theta_{b}}{\partial z^{2}}+\kappa G_{b}A_{b}\left(\frac{\partial V_{b}}{\partial z}-\Theta_{b}\right)+\rho_{b}I_{b}\omega^{2}\Theta_{b}=0,$$

where *V_b_*, *Θ_b_* and *ω* are the displacement in the frequency domain, the rotation angle in the frequency domain and the angular frequency.

The general solutions of *V_b_* and *Θ_b_* can be written as the following formulae:

(5) $$V_{b}=\sum_{j=1}^{4}B_{j}e^{-ik_{\mathrm{bj}}z},$$

(6) $$\Theta_{b}=\sum_{j=1}^{4}\eta_{j}B_{j}e^{-ik_{\mathrm{bj}}z},$$

where $i=\sqrt{-1}$, *B_j_* and *η_j_* are the frequency-dependent coefficients, and *k_bj_* is the transverse wave number.

Substituting Equations (5) and (6) into Equations (3) and (4) leads to

(7) $$k_{b}^{4}-\rho_{b}\left(\frac{1}{E_{b}}+\frac{1}{\kappa G_{b}}\right)\omega^{2}k_{b}^{2}+\left(\frac{\rho_{b}^{2}}{\kappa G_{b}E_{b}}\omega^{2}-\frac{\rho_{b}A_{b}}{E_{b}I_{b}}\right)\omega^{2}=0,$$

from which one can obtain

(8) $$k_{b1}=-k_{b2}=\frac{1}{\sqrt{2}}\sqrt{\rho_{b}\left(\frac{1}{E_{b}}+\frac{1}{\kappa G_{b}}\right)\omega^{2}+\sqrt{4\frac{\rho_{b}A_{b}}{E_{b}I_{b}}\omega^{2}+\frac{\rho_{b}^{2}}{E_{b}^{2}}{\left(1-\frac{E_{b}}{\kappa G_{b}}\right)}^{2}\omega^{2}}},$$

(9) $$k_{b3}=-k_{b4}=\frac{1}{\sqrt{2}}\sqrt{\rho_{b}\left(\frac{1}{E_{b}}+\frac{1}{\kappa G_{b}}\right)\omega^{2}-\sqrt{4\frac{\rho_{b}A_{b}}{E_{b}I_{b}}\omega^{2}+\frac{\rho_{b}^{2}}{E_{b}^{2}}{\left(1-\frac{E_{b}}{\kappa G_{b}}\right)}^{2}\omega^{2}}}.$$

Furthermore, the frequency-dependent coefficient *η_j_* is obtained as

(10) $$\eta_{j}=-\frac{i}{k_{bj}}\left(k_{bj}^{2}-\frac{\rho_{b}}{\kappa G_{b}}\omega^{2}\right),\left(j=1,\;2,\;3,\;4\right).$$

The nodal displacements and rotation angles of the beam element at both ends are denoted by *V_b_*_1_, *Θ_b_*_1_, *V_b_*_2_ and *Θ_b_*_2_. Substituting them into Equations (5) and (6), one can obtain

(11) $${\left[\begin{array}{cc} V_{b} & \Theta_{b} \end{array}\right]}^{T}=N_{b}{\left[\begin{array}{cccc} V_{b1} & \Theta_{b1} & V_{b2} & \Theta_{b2} \end{array}\right]}^{T},$$

where ***N****_b_* is the shape function matrix related to *z* and *ω*, and it can be written as

(12) $$N_{b}=\left[\begin{array}{cccc} e^{-ik_{b1}z} & e^{-ik_{b2}z} & e^{-ik_{b3}z} & e^{-ik_{b4}z}\\ \eta_{1}e^{-ik_{b1}z} & \eta_{2}e^{-ik_{b2}z} & \eta_{3}e^{-ik_{b3}z} & \eta_{4}e^{-ik_{b4}z} \end{array}\right]\times {\left[\begin{array}{cccc} 1 & 1 & 1 & 1\\ \eta_{1} & \eta_{2} & \eta_{3} & \eta_{4}\\ e^{-ik_{b1}L_{b}} & e^{-ik_{b2}L_{b}} & e^{-ik_{b3}L_{b}} & e^{-ik_{b4}L_{b}}\\ \eta_{1}e^{-ik_{b1}L_{b}} & \eta_{2}e^{-ik_{b2}L_{b}} & \eta_{3}e^{-ik_{b3}L_{b}} & \eta_{4}e^{-ik_{b4}L_{b}} \end{array}\right]}^{-1}.$$

The relationships between the shear force *Q_b_*, the bending moment *M_b_*, the displacement *V_b_* and the rotation angle *Θ_b_* in the frequency domain are

(13) $$Q_{b}=\kappa G_{b}A_{b}\left(\frac{\partial V_{b}}{\partial z}-\Theta_{b}\right),$$

(14) $$M_{b}=E_{b}I_{b}\frac{\partial \Theta_{b}}{\partial z}.$$

The shear forces and the bending moments of the beam element at both ends are denoted by *Q_b_*_1_, *M_b_*_1_, *Q_b_*_2_ and *M_b_*_2_. Substituting Equation (11) into Equations (13) and (14) results in

(15) $${\left[\begin{array}{cccc} Q_{b1} & M_{b1} & Q_{b2} & M_{b2} \end{array}\right]}^{T}=S_{b}^{e}\left(\omega \right){\left[\begin{array}{cccc} V_{b1} & \Theta_{b1} & V_{b2} & \Theta_{b2} \end{array}\right]}^{T},$$

where $S_{b}^{e}$ is the 4 × 4 spectral stiffness matrix of the beam element, which is listed in Appendix A.

The spectral stiffness matrix of the tensional element (rod element) of the axially deformed beam can be derived when only the axial force and the axial displacement are considered for the beam. The relationship between the nodal axial forces *F_r_*_1_ and *F_r_*_2_, and the nodal axial displacements *W_r_*_1_ and *W_r_*_2_ in the frequency domain can be written as [41,42,43]

(16) $$\left[\begin{array}{c} F_{r1}\\ F_{r2} \end{array}\right]=S_{r}^{e}\left(\omega \right)\left[\begin{array}{c} W_{r1}\\ W_{r2} \end{array}\right],$$

where $S_{r}^{e}$ is the 2 × 2 spectral stiffness matrix of the tensional element, which has the following form:

(17) $$S_{r}^{e}(\omega )=\left[\begin{array}{cc} S_{r11}^{e} & S_{r12}^{e}\\ S_{r21}^{e} & S_{r22}^{e} \end{array}\right]=\frac{k_{r}E_{b}A_{b}}{\sin\;k_{r}L_{b}}\left[\begin{array}{cc} \cos\;k_{r}L_{b} & -1\\ -1 & \cos\;k_{r}L_{b} \end{array}\right],$$

where $k_{r}=\omega \sqrt{\rho_{b}/E_{b}}$ is the longitudinal wave number.

The spectral element formulation of the beam element with axial deformation can be obtained by assembling the spectral stiffness matrices of the beam and rod elements in Equations (15) and (17). The spectral stiffness matrix $S_{\mathrm{adb}}^{e}$ of an axially deformed beam element has the dimension of 10 × 10, and it consists of $S_{b}^{e}$ along the *x* and *y* axes and $S_{r}^{e}$ along the *z*-axis [43]:

(18) $$S_{\mathrm{adb}}^{e}=\left[\begin{array}{cccccccccc} S_{b11} & 0 & 0 & S_{b12} & 0 & S_{b13} & 0 & 0 & S_{b14} & 0\\ & S_{b11} & 0 & 0 & S_{b12} & 0 & S_{b13} & 0 & 0 & S_{b14}\\ & & S_{r11}^{e} & 0 & 0 & 0 & 0 & S_{r12}^{e} & 0 & 0\\ & & & S_{b22} & 0 & S_{b22} & 0 & 0 & S_{b24} & 0\\ & & & & S_{b22} & 0 & S_{b22} & 0 & 0 & S_{b24}\\ & & & & & S_{b33} & 0 & 0 & S_{b34} & 0\\ & & & & & & S_{b33} & 0 & 0 & S_{b34}\\ & & \mathrm{sym} & & & & & S_{r22}^{e} & 0 & 0\\ & & & & & & & & S_{b44} & 0\\ & & & & & & & & & S_{b44} \end{array}\right].$$

### 2.2. Finite Element Formulation of Thin Plates

For the two thin cover plates, considering only the out-of-plane deformation, the dynamic stiffness matrix $S_{p}^{e}$ of a Kirchhoff plate element is a 12 × 12 matrix, and it can be derived as [41,42]

(19) $$S_{p}^{e}(\omega )=K_{p}^{e}-M_{p}^{e}\omega^{2},$$

(20) $$K_{p}^{e}=\int_{V_{p}{}^{e}}B_{\mathrm{pb}}^{T}R_{\mathrm{pb}}B_{\mathrm{pb}}\;dV,$$

(21) $$M_{p}^{e}=\int_{V_{p}{}^{e}}\rho_{p}N_{p}^{T}N_{p}\;dV,$$

where $K_{p}^{e}$ is the stiffness matrix, $M_{p}^{e}$ is the mass matrix, $V_{p}^{e}$ is the volume of the plate element, ***B****_pb_* is the second-order partial derivative matrix of the shape functions, ***R****_pb_* is the flexural rigidity matrix and ***N****_p_* is the shape function matrix.

For the plate with both the out-of-plane and in-plane deformations, the displacements along the *x*, *y* and *z* axes are denoted by *u_p_*, *v_p_* and *w_p_*. Comparing with the plate, considering only the out-of-plane vibration, including both the out-of-plane and in-plane deformations for the plate, the axial bending component in the stiffness matrix is changed and the transverse shearing component should be added, as *u_p_* and *v_p_* are the functions of *x* and *y*.

The relationships of the strains and displacements of the plate element nodes are written as follows:

(22) $$\varepsilon_{\mathrm{pb}}=B_{pb}d_{p},$$

(23) $$\varepsilon_{ps}=B_{ps}d_{p},$$

where ***ε****_pb_* and ***ε****_ps_* are the bending and shear strain vectors, ***B****_pb_* and ***B****_ps_* are the first-order partial derivative bending and shear matrices of the shape functions and ***d****_p_* is the displacement vector.

The relationships of the stresses and strains can be expressed as

(24) $$\sigma_{\mathrm{pb}}=R_{pb}\varepsilon_{\mathrm{pb}},$$

(25) $$\sigma_{ps}=R_{ps}\varepsilon_{ps},$$

where ***σ****_pb_* and ***σ****_ps_* are the bending and shear stress vectors, and ***R****_pb_* and ***R****_ps_* are the bending and shear modulus matrices.

The dynamic stiffness matrix $S_{p}^{e}$ of the plate element, considering both the out-of-plane and in-plane vibrations, is a 20 × 20 matrix. The forms of Equations (19) and (21) are unchanged, and Equation (20) is changed into the following form:

(26) $$K_{p}^{e}=\int_{V_{p}{}^{e}}B_{\mathrm{pb}}^{T}R_{\mathrm{pb}}B_{\mathrm{pb}}\;dV+\int_{V_{p}{}^{e}}B_{ps}^{T}R_{ps}B_{ps}\;dV.$$

### 2.3. Dynamic Stiffness Matrix of the Metamaterial Sandwich Plate

**Figure 2:**
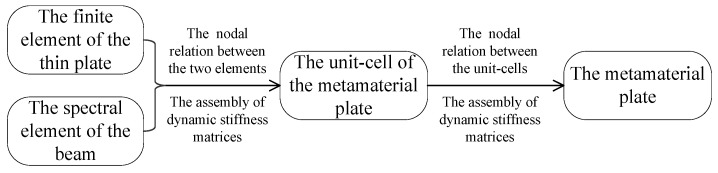
The analysis steps of dynamic stiffness matrix of the metamaterial sandwich plate.

The metamaterial sandwich plate consists of many unit-cells, as shown in Figure 1. The element nodal relationships between the unit-cell of the metamaterial plate, the thin plate and the axially deformed beam are the same as those in Ref. [41]. Figure 2 is the flowchart representing the analysis steps of the dynamic stiffness matrix related to the FE-SEHM. The dynamic stiffness matrix $S_{\mathrm{mp}}^{e}$ of the unit-cell is a 90 × 90 matrix, which can be obtained by connecting the element nodes of the beam and the plates and assembling $S_{\mathrm{adb}}^{e}$ and $S_{p}^{e}$. The dynamic stiffness matrix $S_{\mathrm{mp}}$ of the whole metamaterial sandwich plate can be obtained by connecting the nodes and assembling $S_{\mathrm{mp}}^{e}$. Then, the FRFs of the whole structure can be calculated, and the band-gap properties can be investigated.

## 3. Numerical Results and Discussions

**Figure 3:**
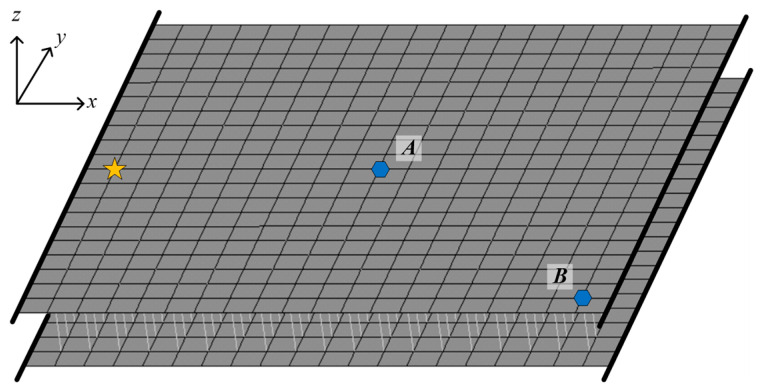
The metamaterial sandwich plate with two opposite clamped edges.

As shown in Figure 3, the metamaterial sandwich plate with periodically distributed axially deformed beam cores as local resonators is clamped at the two opposite edges in the *x*-direction. The time-harmonic forces are applied at the two five-pointed star points (*x* = −0.95 m, *y* = 0 m) of the two cover plates. The pick-up points of the responses of the plate, i.e., the two hexagonal points *A* (*x* = 0 m, *y* = 0 m) and *B* (*x* = 0.95 m, *y* = −1 m), are located at the top cover plate. In the calculations, the structural and material parameters are as follows: For the two face plates: the length along the *x*-axis *a_p_* = 1 m, the width along the *y*-axis *b_p_* = 1 m, the thickness *h_p_* = 0.005 m, the elastic modulus *E_p_* = 70 GPa, the Poisson’s ratio *μ_p_* = 0.33 and the density *ρ_p_* = 2700 kg/m^3^. For the axially deformed beam cores: the length *L_b_* = 0.1 m, the radius *r_b_* = 0.01 m, the elastic modulus *E_b_* = 7.84 MPa, the shear modulus *G_b_* = 2.63 MPa and the density *ρ_b_* = 1225 kg/m^3^. There are 19 × 19 axially deformed beams in total, periodically distributed between the two cover plates.

### 3.1. Validations

**Figure 4:**
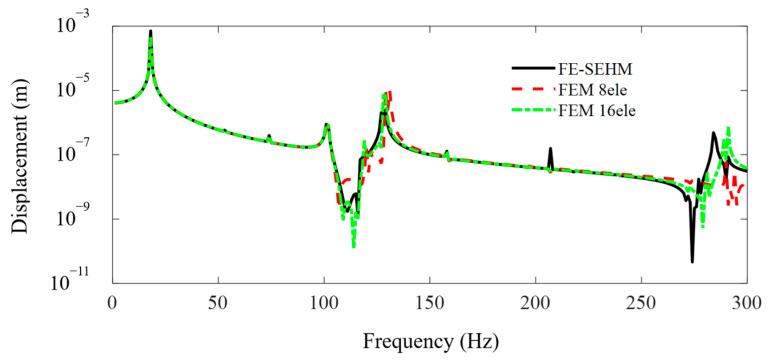
The FRF curves of the metamaterial plate at point A of the cover plate along the *x* axis obtained by the FEM and the FE-SEHM.

**Figure 5:**
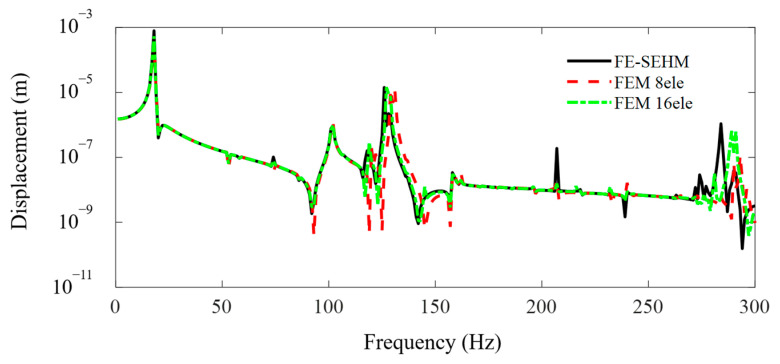
The FRF curves of the metamaterial plate at point B of the cover plate along the *x* axis obtained by the FEM and the FE-SEHM.

The FEM simulations for the metamaterial sandwich plate with periodically distributed axially deformed beam cores are performed using the FEM commercial software and compared with the present developed FE-SEHM. Figure 4 and Figure 5 display the FRF curves of the metamaterial plate at points *A* and *B* of the cover plate along the *x*-axis obtained by the FEM software and the FE-SEHM. In the FEM, each beam is divided into eight or sixteen elements, but in the FE-SEHM, each beam is treated just as one spectral element. It can be clearly seen that with the increase in the element number of the FEM, the results obtained by the FEM gradually converge to those calculated by the FE-SEHM. In the high frequency ranges, the accuracy of the results calculated by the FEM is reduced, with frequency increasing so that and the differences between the results obtained from the FEM and FE-SEHM are not negligible. However, the accuracy of the results calculated by the FE-SEHM is much less sensitive to the frequency. The FE-SEHM can obtain more accurate vibration responses and band-gap characteristics using fewer elements, which validates the feasibility and correctness of the present modeling method.

### 3.2. Band-Gap Characteristics Analysis

**Figure 6:**
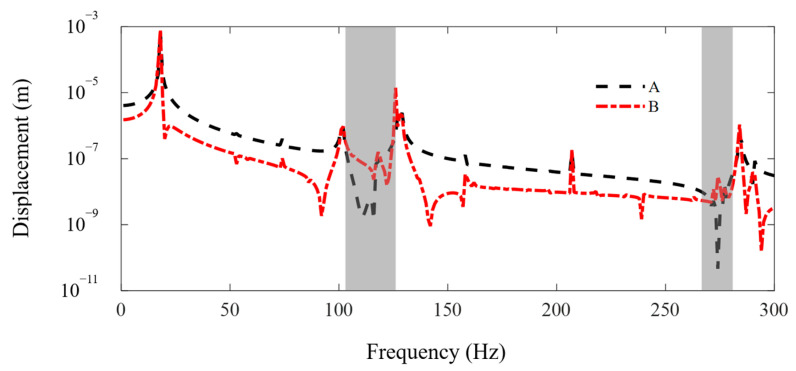
The FRF curves of the metamaterial plate at points A and B of the cover plate along the *x* axis. The dotted line and the dash-dotted line denote the FRF curves of points A and B.

**Figure 7:**
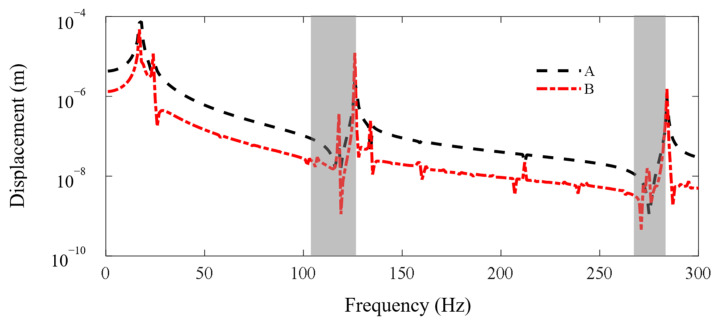
The FRF curves of the metamaterial plate at points A and B of the cover plate along the *y* axis. The dotted line and the dash-dotted line denote the FRF curves of points A and B.

In this subsection, the FE-SEHM is applied to calculate the FRF curves to characterize the band-gap properties of the metamaterial sandwich plate with axially deformed beam cores. Figure 6 and Figure 7 present the FRF curves of the metamaterial plate at points *A* and *B* of the top cover plate along the *x* and *y* axes in the frequency range of 0–300 Hz. There are obviously two band-gaps, i.e., 103–125 Hz and 264–282 Hz, near the first two flexural vibration natural frequencies, 118 Hz and 275 Hz, of the axially deformed beams. The locations where the in-plane vibration band-gaps appear are associated with the natural frequencies of the flexural vibrations of the local resonators. The low band-gap is wider, and the high band-gap is narrower. This is related to the bending mode shapes of the axially deformed beams and the values of the modal participation factors in each order. The vibration of the metamaterial plate in the *xy* plane is coupled with the vibrations of thin plates and beams. For the first-order mode shape, nodal displacements at both ends of the beam are the same, and nodal rotation angles are reversed. For the second-order mode shape, nodal rotation angles at both ends of the beam are the same, and nodal displacements are reversed. Two thin cover plates have corresponding mode shapes. Therefore, the bending modes of the beams can play a leading role and reaction forces can be applied by the beams on the two thin plates to inhibit the vibration propagation in the *xy* plane. Therefore, the vibration can be absorbed and the band-gaps can be formed.

**Figure 8:**
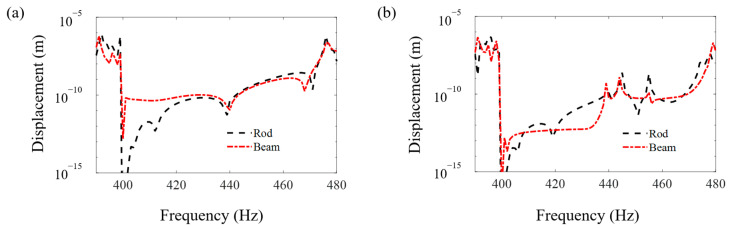
The FRF curves of the metamaterial plates with the beam cores and rod cores near the first axial vibration band-gaps in the *z* direction of the beams at points *A* and *B* of the cover plates. (**a**) Point *A* and (**b**) point *B*.

**Figure 9:**
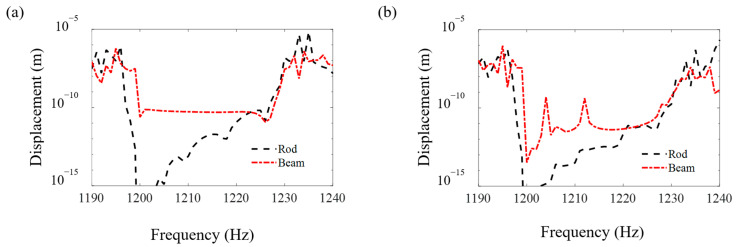
The FRF curves of the metamaterial plates with the beam cores and rod cores near the second axial vibration band-gaps in the *z* direction of the beams at points *A* and *B* of the cover plates. (**a**) Point *A* and (**b**) point *B*.

**Figure 10:**
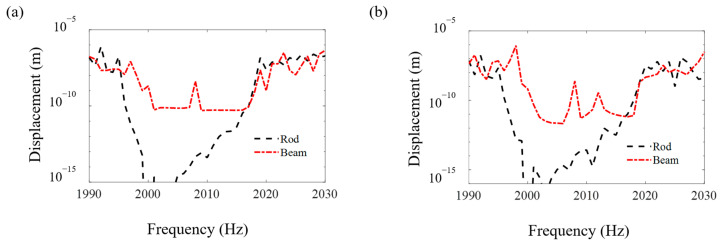
The FRF curves of the metamaterial plates with the beam cores and rod cores near the third axial vibration band-gaps in the *z* direction of the beams at points *A* and *B* of the cover plates. (**a**) Point *A* and (**b**) point *B*.

Figure 8, Figure 9 and Figure 10 compare the FRF curves of the metamaterial plate with axially deformed beam cores near the first three axial vibration band-gaps in the *z* direction of the beams at points *A* and *B* of the cover plate with those of the metamaterial plate with rod cores. Near the odd-order axial vibration natural frequencies, 400 Hz, 1200 Hz and 2000 Hz, of the axially deformed beam, there are three obvious band-gaps, i.e., 400–473 Hz, 1197–1232 Hz and 1996–2019 Hz, in the metamaterial sandwich plates with the beam cores, which are the same as the plates with the rod cores [41].

Furthermore, it is seen in Figure 8, Figure 9 and Figure 10 that the starting and ending frequencies of each order axial vibration band-gap of the beams at points *A* and *B* of the metamaterial sandwich plates with the axially deformed beam cores are the same as those for the sandwich plates with the rod cores. For the metamaterial plates with the beam cores subjected to the axial loads, the transverse displacements are small but not zero, which results in a slight increase in the response amplitudes within the band-gaps compared with the response amplitudes of the plate with rod cores. Compared with the metamaterial sandwich plates with the rod cores, the vibration reduction effect in the band-gaps is declined for the metamaterial plates with the axially deformed beam cores. Consequently, if the transverse displacements of the axially deformed beam cores are ignored, the vibration reduction effect in the metamaterial sandwich plates may be enlarged to a certain extent, which needs great attention in the practical engineering applications.

**Figure 11:**
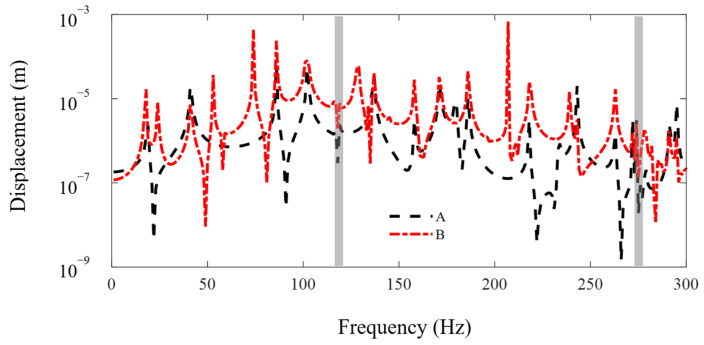
The FRF curves along the *z* axis at points *A* and *B* of the metamaterial plate. The dotted line and the dash-dotted line denote the FRF curves of point *A* and point *B*.

Figure 11 shows the FRF curves along the *z* axis at points *A* and *B* of the metamaterial plate in the frequency range of 0–300 Hz. There are two narrow axial vibration band-gaps in the beams at 118 Hz and 275 Hz, which correspond with the first two flexural vibration natural frequencies in the axially deformed beams, and the effects of the vibration reduction could be ignored. It should be noted that other troughs in the figure are just the anti-resonant peaks and they have nothing to do with the local resonant band-gaps. It is uncoupled between the axial and transverse vibration modes of the beams in the classical axially deformed beam theory. The effect of transverse bending mode on the axial tensional mode is very small and negligible. Therefore, the metamaterial sandwich plates, considering the axial tensional and transverse bending modes of the axially deformed beams, have axially and transversely local resonant band-gaps that are independent with each other and play an important role in reducing the vibrations along the *z* axis and the *x* and *y* axes.

## 4. Conclusions

An elastic metamaterial sandwich plate with axially deformed Timoshenko beam cores, considering both the in-plane and out-of-plane deformations of the face plates, is designed to reduce vibrations by utilizing the band-gap characteristics. Based on the advantages of the FE-SEHM, the dynamic stiffness matrix of the metamaterial sandwich plate is deduced, and the axial and transverse band-gap characteristics of the beam cores are efficiently analyzed. From the investigations, the main conclusions that follow can be drawn:

(1) The FE-SEHM is developed to obtain the dynamic stiffness matrix of the metamaterial sandwich plate with axially deformed beam cores, and the vibration band-gap characteristics can be efficiently characterized by calculating the FRFs of the structure.
(2) Compared with the metamaterial plate with rod cores, the positions and widths of the axial vibration band-gaps of the beams for the metamaterial plate with axially deformed beam cores are the same, but the vibration reduction effect is declined, which should be noticed in the practical applications.
(3) The flexural vibration band-gaps of the beams for the metamaterial sandwich plate along the *x* and *y* axes appear near the flexural vibration natural frequencies of the axially deformed beams, and the lower order band-gaps are wider than the higher order ones.
(4) The flexural vibration band-gaps of the metamaterial sandwich plate can be found uncoupling with the axial vibration band-gaps. The two kinds of band-gaps reduce the vibrations of the plate along the *z* axis and the *x* and *y* axes independently.

## Data Availability

Data available on request due to restrictions e.g., privacy or ethical. The data presented in this study are available on request from the corresponding author.

## Appendix A

The spectral stiffness matrix $S_{b}^{e}$ of the beam element in Equation (15) is expressed as

(A1) $$S_{b}^{e}=\left[\begin{array}{cccc} S_{b11} & S_{b12} & S_{b13} & S_{b14}\\ & S_{b22} & S_{b23} & S_{b24}\\ & & S_{b33} & S_{b34}\\ sym & & & S_{b44} \end{array}\right],$$

where

$S_{b11}=S_{b33}=-iE_{b}I_{b}C_{1}C_{3}[(e_{1}^{2}-1)(e_{3}^{2}+1)\eta_{1}-(e_{1}^{2}+1)(e_{3}^{2}-1)\eta_{3}](k_{1}\eta_{1}-k_{3}\eta_{3})$,

$S_{b12}=-S_{b34}=iE_{b}I_{b}C_{1}C_{3}\{(e_{1}^{2}-1)(e_{3}^{2}-1)(k_{1}\eta_{3}+k_{1}\eta_{3})-[(e_{1}^{2}+1)(e_{3}^{2}+1)-4e_{1}e_{3}](k_{1}\eta_{1}+k_{3}\eta_{3})\}$,

$S_{b13}=2iE_{b}I_{b}C_{1}C_{3}[(e_{1}^{2}-1)e_{3}\eta_{1}-(e_{3}^{2}-1)e_{1}\eta_{3}](k_{1}\eta_{1}-k_{3}\eta_{3})$,

$S_{b14}=-S_{b23}=2iE_{b}I_{b}C_{1}C_{3}(e_{1}-e_{3})(e_{1}e_{3}-1)(k_{1}\eta_{1}-k_{3}\eta_{3})$,

$S_{b22}=S_{b44}=iE_{b}I_{b}C_{2}C_{3}[-(e_{1}^{2}+1)(e_{3}^{2}-1)\eta_{1}+(e_{1}^{2}-1)(e_{3}^{2}+1)\eta_{3}]$,

$S_{b24}=2iE_{b}I_{b}C_{2}C_{3}[(e_{1}^{2}-1)e_{1}r_{1}-(e_{3}^{2}-1)e_{3}r_{3}]$,

$C_{1}={\left(1-\frac{\rho_{b}I_{b}}{\kappa G_{b}A_{b}}\omega^{2}\right)}^{-\frac{1}{2}}$, $C_{2}={\left[4+\frac{\rho_{b}I_{b}}{E_{b}A_{b}}{\left(1-\frac{E_{b}}{\kappa G_{b}}\right)}^{2}\omega^{2}\right]}^{\frac{1}{2}}$,

$C_{3}=\sqrt{\frac{\rho_{b}A_{b}}{E_{b}I_{b}}}\cdot \frac{i\omega }{\left\{2\eta_{1}\eta_{3}[(e_{1}^{2}+1)(e_{3}^{2}+1)-4e_{1}e_{3}]-(\eta_{1}^{2}+\eta_{3}^{2})(e_{1}^{2}-1)(e_{3}^{2}-1)\right\}}$,

$e_{1}=e^{-ik_{b1}L_{b}}$, $e_{3}=e^{-ik_{b3}L_{b}}$.
